# Stronger Correlation between Interleukin 18 and Soluble Fas in Lupus Nephritis Compared with Mild Lupus

**DOI:** 10.1155/2013/850851

**Published:** 2013-03-14

**Authors:** Mohammad Reza Hatef, Maryam Sahebari, Zahra Rezaieyazdi, Mohammad Reza Nakhjavani, Mahmoud Mahmoudi

**Affiliations:** ^1^Rheumatology, Rheumatic Diseases Research Center (RDRC), School of Medicine, Mashhad University of Medical Sciences, Mashhad, Iran; ^2^Rheumatology, Rheumatology Department, School of Medicine, Tabriz University of Medical Sciences, Tabriz, Iran; ^3^Immunology, Immunology Research Center, School of Medicine, Mashhad University of Medical Sciences, Mashhad, Iran

## Abstract

Lupus nephritis (LN) is a major cause of morbidity in patients with systemic lupus erythematosus (SLE). Several cytokines and apoptotic markers such as IL-18 and soluble Fas (sFas) have been assumed to play a role in the pathogenesis of LN. Previous studies confirmed that serum concentrations of sFas and IL-18 are increased in SLE. However, only a few studies have suggested a possible correlation between IL-18 and sFas. This study was planned to continue our previous study on the correlation between those markers to evaluate this correlation in LN. Thirty-two patients with only LN and 46 patients without any major organ involvement participated in this study. SLEDAI score (except for scores related to nephritis) was the same in these two groups. In both groups, patients with any other major organ involvement were excluded. We found a significant rise in the serum concentrations of sFas (*P* = 0.03) and IL-18 (*P* = 0.02) in patients with proteinuria compared to those without it. This study showed that the correlation between sFas and IL-18 in LN (*P* < 0.001, *r*_*p*_ = 0.5) is significantly stronger than it is in mild SLE (*P* < 0.001, *r*_*p*_ = 0.4) with similar nonrenal SLEDAI score (*P* = 0.032, *z* = 1.85). Between these two serum markers, sFas is the only predictor of proteinuria.

## 1. Introduction 

Lupus nephritis is a serious complication of SLE. Proteinuria is the most frequently observed abnormality in lupus nephritis [1, 2]. Although the precise etiology of LN is not entirely known, several factors have been proposed in the initiation and progression of LN. Two important factors that are suggested to be involved in that are apoptosis imbalance [3] and overproduction of several cytokines like IL-18 [4].

 Researchers have emphasized the pathogenic function of IL-18 and Fas/Fas ligand pathway in autoimmune-related diseases like lupus [5–7]. Besides, recent evidence suggests that IL-18- and Fas-mediated apoptosis may relate to each other by the proapoptotic effects of IL-18. IL-18 is able to enhance Fas/Fas ligand expression in specific cells [8, 9]. 

Fas (Apo/1-CD95) and its ligand belong to the tumor necrosis factor/nerve growth factor superfamily [10–16]. IL-18, a TNF-*α* inducer and Fas/Fas ligand expressor, is a crucial factor for the autoimmune process [5, 8, 9, 17–19]. Although the role of IL-18 and sFas has been elucidated separately, in the pathogenesis of LN [1, 4, 20], there is little evidence about the correlation between sFas and IL-18 in autoimmune diseases. Only a few studies mentioned that infections could raise serum sFas and IL-18 concentrations through increasing and/or enhancing apoptotic turnover of defensive cells [21–26]. Very few studies about autoimmune diseases have addressed this correlation too. Chen et al. [27] demonstrated the influence of IL-18 on the apoptosis of peripheral blood lymphocytes in adult onset Still's disease (AOSD), SLE, and healthy participants. In our previous study, we also demonstrated that sFas and IL-18 rise in correlation with disease activity in lupus [28].

A large and growing body of literature expressed the role of IL-18 and sFas in lupus nephritis apart from each other [4, 18, 20, 29–37], and some investigators referred to the local production of IL-18 in glomeruli resulting in local effects in the pathogenesis of LN [38]. However, far too little attention has been paid to the correlation between these two serum markers in LN. 

To continue our previous study, in this paper, we carefully examined the correlation between sFas and IL-18 serum concentrations in lupus nephritis compared with mild lupus. The main question in the current study was whether the correlation between sFas and IL-18 in LN is stronger than that correlation in mild lupus. For this purpose, we designed this study by the selection and comparison of two groups of patients including lupus nephritis patients without any other major organ involvement and mild lupus patients including those without any major organ involvement. 

## 2. Materials and Methods

### 2.1. Patients and Controls

This is a prospective case-control, cross-sectional study in which seventy-eight SLE patients including 75 (96.2%) women and 3 (3.8%) men participated. Thirty-two (41%) patients had proteinuria more than 500 mg in a 24-hour urine collection (case or severe SLE group), and 46 (59%) patients had no kidney involvement as defined by normal urinary sedimentation, creatinine clearance more than 80%, without previous history of renal involvement (control or mild SLE group). All patients have been diagnosed SLE by fulfilling at least four criteria of the American College of Rheumatology (ACR) revised criteria for SLE [34]. Renal involvement in this study was defined as proteinuria more than 500 mg in a 24-hour urinary collection sample, nephritic hematuria or pyuria, or GFR less than 80%. A provided checklist recorded patients' demographic data including important laboratory parameters and medications. SLEDAI questionnaire was completed for each participant. Pregnant or postpartum women, patients with past or present history of malignancy, concurrent infection, recent trauma, smoking or addiction, overlap syndromes, chronic renal failure, and other systemic problems not related to SLE like a history of hepatitis or liver disease were excluded from the study. Since reduction in glomerular filtration rate (GFR) increases serum sFas concentrations [35], we also excluded all patients with the GFR less than 80%. Thus, the aim of this study was the evaluation of sFas and IL-18 serum levels in the lupus renal involvement. Patients with other major organ involvements at the sampling time, like heart or central nervous system involvement, vasculitis, and psychosis were also excluded. Sera was obtained from patients and kept frozen at −20°C prior to measurements of IL-18 and sFas as described below. All participants signed an informed consent. This study was approved by the Mashhad University of Medical Sciences ethic committee.

### 2.2. IL-18 and Soluble Fas (CD95/Apo-1) ELISA

All samples were coded and the laboratory technicians were kept blind about the cases and controls. sFas concentrations were detected using a sAPO-1/FAS BMS245 ELISA kit (Bender Medsystems, Austria) according to the manufacturer's instructions. The overall intra- and interassay coefficients of variation in this study were 4.5% and 3.1%, respectively. IL-18 concentrations were measured with BMS267 ELISA kit (Bender Medsystems, Austria) according to the manufacturer's instructions. The overall intra- and interassay coefficients of variation in this study were 6.5% and 8.1%, respectively.

### 2.3. Statistical Analysis

The statistical analyses were performed with the SPSS 11.5 program (SPSS Inc., Chicago, IL, USA). Values are reported as mean ± SD for normally distributed variables and median with interquartile range (IQR) for others. To assess variables that are not normally distributed, “Kolomogrov-Smirnov” test was used. Patient's demographics and clinical characteristics were analyzed using “student's *t*-test” for continuous variables, “Mann-Whitney test” and “Kruskal-Wallis test” for nonparametric variables, and “chi-square test” for categorical variables. The correlation coefficient was calculated according to Pearson and Spearman's rank correlation coefficient test based on the data type. Multivariate logistic regression analyses were performed to evaluate the association between IL-18 and sFas serum levels with the existence of proteinuria. “Significance of the difference between two correlation coefficients” was applied to estimate the power of correlation between sFas and IL-18 among case and control groups.

## 3. Results

### 3.1. Baseline Analysis

One-sample Kolmogorov-Smirnov test was used to evaluate distribution of age, IL-18, sFas, C3, C4, ESR, anti-dsDNA, and 24-hour urine protein excretion parameters in three groups including all participants, severe lupus, and mild lupus separately.  Table 1 presents distribution of these parameters in patients. 

### 3.2. Clinical Characteristics

Seventy-eight lupus patients enrolled in this study. Thirty-two of them had proteinuria. The mean duration of disease was 4 years. The median with interquartile range (IQR) of 24-hour urine protein excretion was 1.2 (0.6–2) gram. The mean level of creatinine in the patients was 0.7 (±0.2) mg/dL. The mean (± SD) or median (IQR) of the age, IL-18, sFas, C3, C4, ESR, and anti-dsDNA in all participants and the statistical difference of these parameters between the 2 groups are shown in Table 1. 

Twenty-four patients with proteinuria underwent renal biopsy. Among these, 20 cases had class IV LN, 2 had class V LN, and 2 cases had class II LN.

As Table 1 shows, there were not any significant difference in important laboratory parameters like a rise in anti-dsDNA and ESR or a decrease in C3, C4, thrombocytopenia, lymphopenia, and hemolytic anemia between the two groups. The mean of SLEDAI score without renal involvement criteria (proteinuria, hematuria, and pyuria) in LN patients was 8.1 ± 5.7 and in the control group it was 10.9 ± 7.2. There was not any statistical difference in nonnephrotic SLEDAI criteria between the two groups (*P* = 0.6, *t* = −1.8).

### 3.3. sFas and IL-18 in the Two Groups of Patients with and without Proteinuria

**Figure 1:**
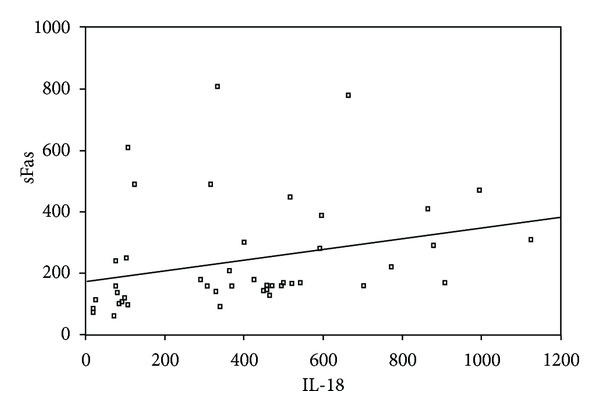
Correlation between serum values of sFas and IL-18 in patients with proteinuria (*P* < 0.001, *r*_*p*_ = 0.5).

**Figure 2:**
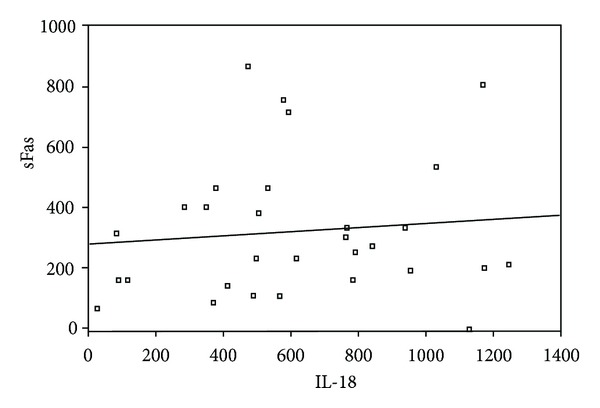
Correlation between serum values of sFas and IL-18 in patients without proteinuria (*P* < 0.001, *r*_*p*_ = 0.4).

Serum levels of sFas (*P* = 0.03) and IL-18 (*P* = 0.02) in the patients with proteinuria were significantly higher than patients without proteinuria (Table 1). We found a significant positive correlation between serum levels of sFas and IL-18 in patients with proteinuria (*P* < 0.001, *r*_*p*_ = 0.5) (Figure 1). There was also a positive correlation between these two parameters in patients without proteinuria (*P* < 0.001, *r*_*p*_ = 0.4) (Figure 2). After applying significance of the difference between two correlation coefficients, correlation coefficient of sFas and IL-18 in the patients with proteinuria was found greater than those without it (*z* = 1.85, *P* = 0.032, one-tailed). As we mentioned above, these two groups did not show any statistical difference in nonnephrotic SLEDAI scores. 

### 3.4. Medications

Sixty-six of our patients were treated with prednisolone. The mean IQR dose of prednisolone was 10 (5–30) mg/d. There was no correlation between sFas (*P* = 0.42, *r* = 0.1) and IL-18 (*P* = 0.33, *r* = 0.12) serum levels and prednisolone dosage (Spearman's rank correlation test). Forty-eight percent of our patients received cytotoxic drugs as follows: 38% cyclophosphamide, 34% azathioprine, 14% mycophenolate mofetil, 12% methotrexate, and 2% a combination of cyclophosphamide and mycophenolate mofetil. We did not find any significant difference in serum levels of sFas (*P* = 0.16, Chi = 1.9) and IL-18 (*P* = 0.2, Chi = 1.5) between the patients who received cytotoxics and those who did not (Kruskal-Wallis test) in total patients. 

### 3.5. Multiple Logistic Regression Analysis

Multiple logistic regression analysis was conducted to determine the impressive role of IL-18 and sFas on proteinuria. The analysis identified sFas (O.R. = 1.003; 95% CI, 1.002–1.005; *P* = 0.029) as a significant predictor of proteinuria in lupus nephritis.

## 4. Discussion

 It is well known that overproduction of several cytokines and imbalance of apoptotic pathways play a significant role in the pathogenesis of LN [4–6]. The results of this study were in line with our previous study, which revealed a strong positive correlation between serum levels of sFas and IL-18 in lupus [12–28] and other studies that demonstrated the impressive action of IL-18 [4, 5, 32, 36–38] and sFas [3, 31, 32, 39] in LN. Although we found this positive correlation in both patients with and without proteinuria, this correlation was stronger in LN. Moreover, in this study, patients were selected in a way that nonrenal major organ involvements were excluded and the mean of nonrenal SLEDAI scores did not show any statistical difference between the two groups. Therefore, the differences between sFas and IL-18 serum values in the two groups are most likely justified by renal involvement. According to our literature review, there was not any noticeable data available on the correlation between sFas and IL-18 in LN. Considering this correlation, most of the previous studies that pointed to it were conducted on infections [21–26]. In addition, we found that sFas is a stronger predictor for LN than IL-18. This result may be justified by observations which showed that local production of IL-18 in glomeruli is more important than systemic IL-18 for induction of LN [18, 38]. Faust et al. showed that in MRL-Faslpr mice with autoimmune lupus nephritis, renal tubular epithelial cell-derived interleukin-18 upregulation correlates with disease activity [30]. Furthermore, these positive correlations were consistent despite immunosuppressive therapy. In other words, it can be supposed that this correlation may be stronger in untreated patients, although this needs to be verified with further studies.

However, our study is a cross-sectional study with a limited number of patients, which does not have enough power to confirm the aforementioned hypothesis. In conclusion, one can assume that a balance among various immunologic pathways such as apoptotic pathways, cytokine production and synthesis of their soluble receptors or inhibitors, predict the natural course of a disease. Based on our findings, we propose that in lupus nephritis, IL-18 and sFas have interrelated effects on proteinuria. Further research to investigate the association of IL-18 and sFas and their exact role in induction or maintaining of lupus nephritis is required.

## 5. Conclusions 

In brief, the current study illustrated that serum values of sFas and IL-18 are significantly higher in lupus patients with proteinuria compared with patients without proteinuria. In addition, the correlation between sFas and IL-18 is significantly stronger in LN patients compared to patients without LN. Moreover, sFas serum values are better predictors than IL-18 for proteinuria. The stronger correlation between sFas and IL-18 in LN compared with mild lupus emphasizes the important pathogenic role of these two markers in kidney damage. 

## Figures and Tables

**Table 1:** Important demographic and laboratory data of the patients.

Variables	Total patients (*n* = 78) mean (±SD) or IQR*	Patients without proteinuria (*n* = 46) mean (±SD) or IQR	Patients with proteinuria (*n* = 32) mean (±SD) or IQR	*P* value (patients with and without proteinuria)
Age (years) range	30.14 (±9.5) 15–62	28.98 (±6.8) 20–43	29.47 (±8.9) 15–62	0.6**
C3 (mg/dL) range	41 (30.75–97) 5–163	41 (33–100) 5–163	62.44 (±41.4) 5–150	0.5**
C4 (mg/dL) range	14 (27–10) 4–53	15 (10–32) 5–35	18.41 (±12.5) 4–49	0.5**
Anti dsDNA range	2 (1–7) 1–30	4.43 (±5.4) 1–30	1.5 (1–8) 1–15	0.6***
Cr (mg/dL)	0.77 (±26)	0.78 (±0.26)	0.77 (±0.26)	0.82**
ESR (mm/h) range	25 (18–54) 2–150	25 (17–47) 4–119	48.81 (±39.4) 2–150	0.1***
IL-18 (pg/mL) range	479 (287–775) 20–1245	399.23 (±287.5) 20–1125	615.57 (±342.6) 25–1245	0.02**
sFas (pg/mL) range	210 (148–195) 1–2260	168 (134–292) 61–810	409.38 (±416.3) 1–2260	0.03**
Number of Lymphopenic patients (%)	27 (34.6)	17 (36.9)	10 (31.25)	0.60****
Number of thrombocytopenic patients (%)	9 (11.53)	5 (10.86)	4 (12.5)	1****
Number of patients with hemolytic anemia (%)	3 (3.8)	2 (4.34)	1 (3.1)	1****

*Normally distributed are presented by mean (± SD), not normally distributed data are presented by interquartile (IQR) range.

** Student's *t*-test.

***Mann-Whitney test.

****Chi-square test.
